# Deciphering the functional role of spatial and temporal muscle synergies in whole-body movements

**DOI:** 10.1038/s41598-018-26780-z

**Published:** 2018-05-30

**Authors:** Ioannis Delis, Pauline M. Hilt, Thierry Pozzo, Stefano Panzeri, Bastien Berret

**Affiliations:** 1 School of Biomedical Sciences, University of Leeds, Leeds, LS2 9JT United Kingdom; 2 Department of Biomedical Engineering, Columbia University, New York, NY 10027 USA; 3 INSERM, U1093, Cognition Action Plasticité Sensorimotrice, Universite de Bourgogne, Dijon, France; 4 Fondazione Istituto Italiano di Tecnologia, Centro di Neurofisiologia traslazionale c/o sezione Fisiologia Umana Via Fossato di Mortara, 17-19 44121 Ferrara, Italy; 5 Neural Computation Laboratory, Center for Neuroscience and Cognitive Systems@UniTn, Istituto Italiano di Tecnologia, Via Bettini 31, 38068 Rovereto, (TN) Italy; 6 CIAMS, Univ. Paris-Sud, Université Paris-Saclay, 91405 Orsay, Cedex France; 7 CIAMS, Université d’Orléans, 45067 Orléans, France; 8 Institut Universitaire de France (IUF), Paris, France

## Abstract

Voluntary movement is hypothesized to rely on a limited number of muscle synergies, the recruitment of which translates task goals into effective muscle activity. In this study, we investigated how to analytically characterize the functional role of different types of muscle synergies in task performance. To this end, we recorded a comprehensive dataset of muscle activity during a variety of whole-body pointing movements. We decomposed the electromyographic (EMG) signals using a space-by-time modularity model which encompasses the main types of synergies. We then used a task decoding and information theoretic analysis to probe the role of each synergy by mapping it to specific task features. We found that the temporal and spatial aspects of the movements were encoded by different temporal and spatial muscle synergies, respectively, consistent with the intuition that there should a correspondence between major attributes of movement and major features of synergies. This approach led to the development of a novel computational method for comparing muscle synergies from different participants according to their functional role. This functional similarity analysis yielded a small set of temporal and spatial synergies that describes the main features of whole-body reaching movements.

## Introduction

Human motor control has been hypothesized to rely on a limited set of building blocks, termed muscle synergies, motor primitives or more generically modules^1–7^. A key assumption of this hypothesis is that, in order to generate and execute movement, the central nervous system (CNS) selectively codes motor task parameters via the activation of certain muscle synergies^8,9^. In other words, the brain is assumed to control a small set of task-related variables and trigger low-dimensional motor commands which take the form of synergy activations^10,11^. This combined recruitment of muscle synergies gives rise to patterns of muscle activity that are necessary for the accomplishment of the desired motor task^12,13^. Importantly, understanding how task parameters are actually encoded within the muscle synergy framework would provide useful insights into the hierarchical organization of human motor control^8,14,15^. In this study, we develop a computational approach to probe the encoding of various movement features from distinct muscle synergies and investigate whether the type and shape of muscle synergies relates to the spatiotemporal characteristics of the investigated set of movements.

To this aim, we collect a remarkably large electromyographic (EMG) dataset (30 muscles) during performance of a rich set of whole-body goal-directed movements (72 distinct point-to-point movements), which allows us to investigate the dependence of modular structures on various task features. We then characterize the modular structure of the recorded muscle activity using a modularity model that separates the spatial and temporal aspects of muscle patterns. More precisely, we use a space-by-time decomposition which hypothesizes the existence of invariant temporal and spatial synergies that are combined by scalar activations to execute the desired movement on each trial^16,17^. In this model, temporal synergies represent stereotypical temporal activation profiles that are shared across muscles whereas spatial synergies represent fixed balances of muscle activity at any point in time.

Importantly, here we link the modular decomposition to its functional role, i.e. task performance, by asking what task information is carried by each one of the identified synergies (spatial or temporal). Conceptually, this bridges two different views on muscle synergies, i.e. the building-block approach^1^ with the task-dynamic approach^18^. In practice, to quantify the information about the task contained in the identified synergies, we employ a multivariate decoding and information theoretic analysis^19–21^. Unlike previous work which assessed the task discriminative power of a complete modular decomposition^16,22,23^, here we aim to evaluate the decoding ability of each synergy independently. Hence, by mapping each synergy to a specific task parameter, we dissect its functional role and quantify its distinct contribution to task performance.

We then capitalize on this approach to compare synergies between different subjects based on their functional role as represented by the task parameters they encode rather than their muscle activation profile which may vary significantly across subjects^24–27^. The core idea behind this method is that synergies which are different in muscle composition or temporal profile, can be clustered as similar if they relate to the same task parameters, i.e. they can be considered as functionally equivalent if they have similar roles in task performance. Such considerations of motor equivalence trace back to foundational studies in motor control^2,18,28^. Here our computational method draws upon this conceptual work in conjunction with recent analytical developments in muscle synergy identification from EMG signals^16,29,30^ and the assessment of synergy decompositions in task space^10,14,18,22,31^. Hence, we propose a novel functional similarity analysis that serves to cluster functionally similar synergies across individuals. We identify a small set of temporal and spatial synergies that have complementary task coding functions consistently across subjects and experimental sessions. On the whole, our study provides a detailed analytical account and interpretation of the functional significance of spatial and temporal muscle synergies.

## Results

In the following, we first identified the temporal and spatial muscle synergies that underlie the generation of muscle activation patterns during whole-body pointing. Then, we assessed the encoding of task parameters from the space-by-time synergy activations and investigated whether this modular scheme reflects the desired task-specific motor behavior in single trials. This analysis also characterized the functional role of each synergy with respect to the performed movement and related the function of each synergy with the muscles it activates. Finally, we examined the functional similarity of the extracted synergies across subjects and determined a small set of spatial and temporal synergies whose single-trial activations encode complementary task parameters and describe the muscle activity that is necessary for the distinct representation of the whole-body pointing movements.

### Space-by-time modular decomposition of muscle activity

**Figure 1:**
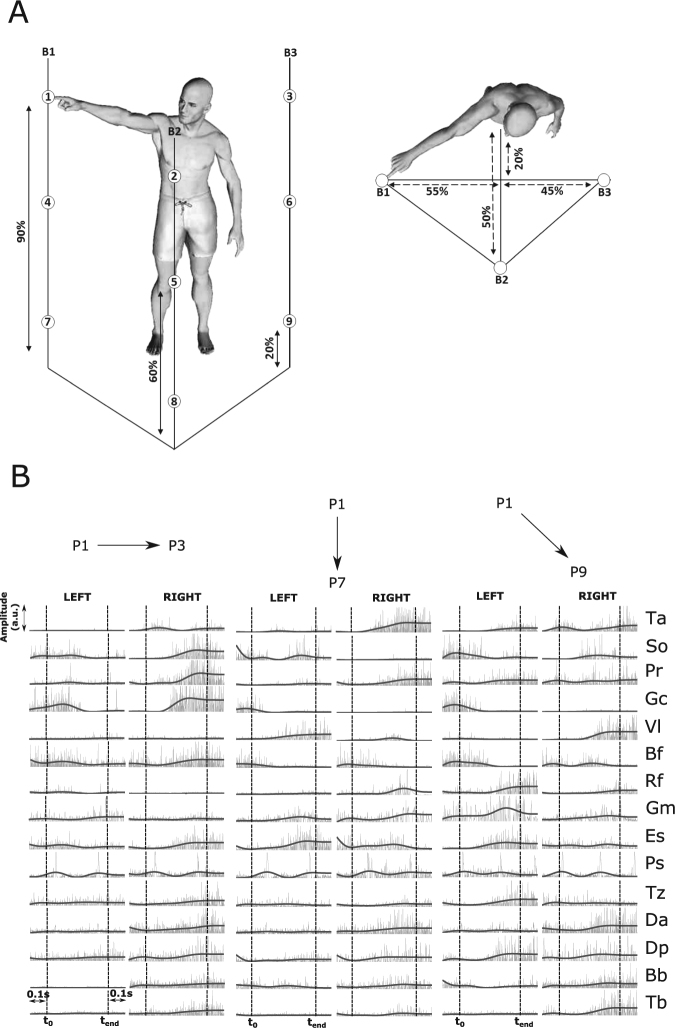
Experimental task and EMG recordings. (**A**) Illustration of the experimental protocol. Placement of the nine targets on three supporting bars (B1, B2, B3, three targets on each vertical bar) is based on the subject’s height (shown as percentages in figure). Subjects performed point-to-point movements between all pairs of targets (a total of 72 movements) and repeated each movement 30 times. (**B**) Raw muscle activation data (normalized to the maximal activation of each muscle) of three trials in which the example subject performed a horizontal leftward movement (P1–P3), a vertical downward movement (P1–P7) and a diagonal leftward and downward movement (P1–P9). The recorded EMG activity of 30 muscles, normalized in amplitude (across the whole experiment for each muscle), are plotted from time *t*_0_ − 0.1 sec to time *t*_*end*_ + 0.1 sec. Raw EMG recordings are shown in gray and filtered EMG signals, which are input to the synergy extraction algorithm, are shown in black. *t*_0_ and *t*_*end*_ (shown as dotted vertical lines) are the movement onset and offset times.

We recorded muscle (electromyographic - EMG) activity from 30 muscles spanning the whole body while four participants performed point-to-point movements between all pairs of 9 targets placed on three vertical bars (B1-left, B2-middle, B3-right) on three different heights (top, middle, bottom) - see Fig. 1 for an illustration of the experimental setup. We then applied the sNM3F algorithm to the (pre-processed) EMG recordings of each participant to extract a space-by-time representation of the single-trial muscle activity during performance of the 72 distinct whole-body pointing movements defined in the experimental protocol. We found that the EMG patterns of all four participants were composed of four temporal synergies (*K* = 4), whereas a different number of spatial synergies was identified across participants (E1: *N* = 4, E2: *N* = 6, E3: *N* = 7, E4: *N* = 5). To evaluate the plausibility of the resulting space-by-time decompositions, we quantified a) how well they approximate the original EMG recordings and b) how well they discriminate in single trials the 72 performed movements. We found that the identified decompositions achieved on average across subjects (mean ± sem) a VAF value of 68 ± 5% and decoding performance of 86 ± 1%. Importantly, these space-by-time decompositions carried all the movement discrimination power afforded in the modular space as no statistically significant gain (*p* < 0.05) in decoding was obtained when adding more (spatial or temporal) synergies. We also refer to^32^ for a thorough decoding performance comparison of the obtained space-by-time decompositions with non-modular representations of the recorded EMG signals.

**Figure 2:**
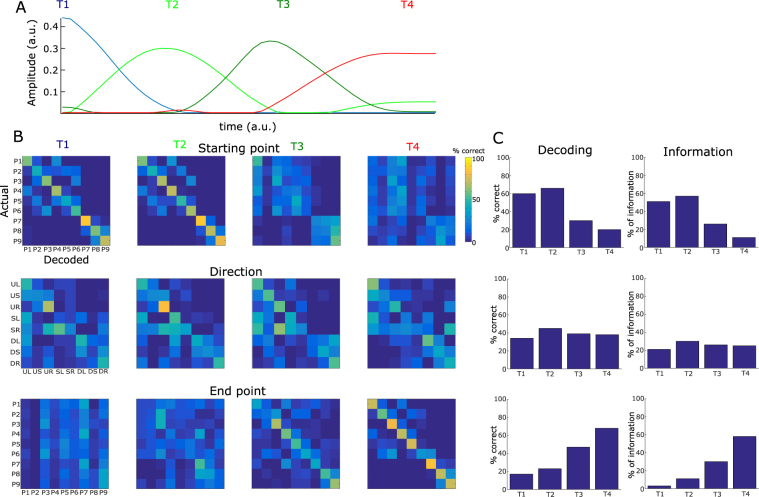
Task coding function of temporal muscle synergies. (**A**) The four temporal synergies extracted from the single-trial EMG data of an example subject. The four synergies are successive in time covering the full movement duration. (**B**) Confusion matrices illustrating how well each one of the four temporal synergies (organized in rows) can discriminate the starting point (top), direction (middle) and endpoint of the movement. (**C**) Percentages of correctly decoded trials (% correct) and percentages of the information about each one of the three “temporal” task parameters (starting point, direction and endpoint) carried by the four temporal synergies.

The temporal synergies of the space-by-time decomposition are time-varying patterns of muscle activity representing the stereotypical temporal profiles of activations shared across muscles. The four temporal synergies extracted from the data of an example participant are shown in Fig. 2A. Each synergy represents a burst of muscle activity and the four bursts are consecutive in time spanning the whole movement duration. The first temporal synergy is active at the time of movement onset suggesting that it partly comprises tonic muscle activity that stabilizes the participant’s posture while pointing to the starting target and the last temporal synergy is active after the end of the movement suggesting that it partly comprises tonic muscle activity that stabilizes the participant’s posture while pointing to the end target. The other two synergies consist of bursts of muscle activity during the transient part of the movement, thus they primarily comprise phasic muscle activity.

**Figure 3:**
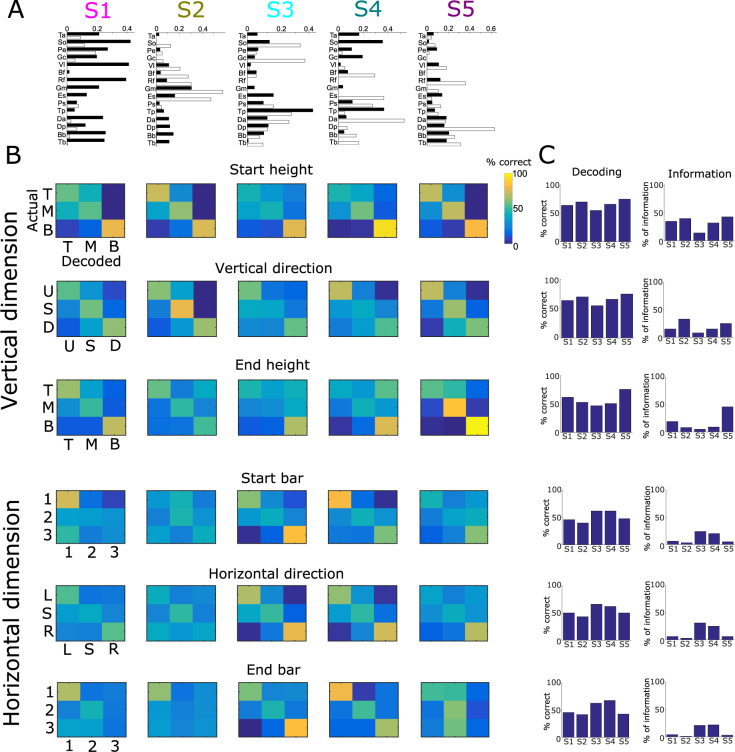
Task coding function of spatial muscle synergies. (**A**) The five spatial synergies extracted from the single-trial EMG data of an example subject as vectors of muscle activation levels. Each of the five synergies comprises muscles from the whole body and both hemobodies (left hemibody muscles shown in white and right hemibody muscles in black). (**B**) Confusion matrices illustrating how well each one of the five spatial synergies (organized in rows) can discriminate task parameters describing the vertical dimension of movement (top three rows - starting height, vertical direction and end height) and task parameters describing the horizontal dimension of movement (bottom three rows - starting bar, horizontal direction and end bar). (**C**) Percentages of correctly decoded trials (% correct) and percentages of the information about each one of the “spatial” task parameters carried by the five spatial synergies.

The spatial synergies are vectors of muscle activity levels representing fixed balances of muscle activations at any point in time. The five spatial synergies of the example participant are shown in Fig. 3A. Each synergy comprises activation of muscles over the entire body including upper and lower limbs and both hemibodies. Hence, the extracted spatial synergies do not separate specific body parts which suggests that they represent functional groupings of muscle activations rather than purely anatomical constraints or couplings resulting from cross-talk from neighboring muscles in EMG recordings. In particular, the first spatial synergy comprises activations of muscles spread across the body primarily in the left hemibody. The second synergy activates primarily upper leg and trunk muscles in the right hemibody, the third and fourth synergies activate mainly postural lower leg muscles (in the right hemibody for the third synergy, in the left hemibody for the fourth synergy) as well as upper body and arm muscles, and the fifth synergy activates primarily muscles of the right (dominant) arm.

### Functional role of temporal muscle synergies

We next investigated what aspects of the task are described by the activation of temporal synergies. To this end, we performed decoding analyses, i.e. we used the activation coefficients combining each temporal synergy with all spatial synergies to decode task parameters and plotted the results in the form of confusion matrices. The decoding results for the temporal synergies of the example subject (E4) are shown in Fig. 2B where each column shows the confusion matrix of a different temporal synergy and each row the decoding of a different task parameter.

Single-trial activations of the first temporal synergy characterize the movement starting point (P1, …, P9, 60% correct decoding - chance level is 11% for all 9-wise discriminations performed in this section and 1.61 bits of information - 51% of the maximum information that is 3.17 bits, see top left confusion matrix in Fig. 2B) as they discriminate the height from which the movement started (78% correct decoding on average, 0.91 information bits out of 1.59 bits maximum information for 3-wise discriminations, i.e. 57% of the maximum information, see Fig. 2C) and the starting bar (1, 2 or 3, 73% correct decoding, 0.59 bits - 37% of information). Bottom starting targets were more reliably discriminated than middle and top (98% vs 70% and 68% respectively). The second temporal synergy also describes the starting target (66% correct decoding, 1.81 bits - 57%) as well as the movement direction (45% correct decoding, 0.89 bits - 30%, maximum information is 3 bits for this 8-wise discrimination). Hence, the second temporal synergy characterizes the movement initiation (starting target and direction) and more specifically whether the subject moved their arm upward, downward or stayed at the same height (70% correct decoding, 0.49 bits - 31%) and whether they moved leftward, rightward or stayed at the same bar (57% correct decoding, 0.27 bits - 17%). In particular, this synergy contributes most to the discrimination of upward movements (76%) and rightward movements (82%). The third temporal also contributes to the discrimination of the movement direction (39% correct decoding, 0.79 bits - 50%) by discriminating mostly the downward movements (72% correct decoding) from all others. It also distinguishes partly the end target (47% correct decoding, 0.95 bits - 60%), though the end target is decoded best by the fourth temporal synergy (68% on average, 76% correct decoding of end height, 78% correct decoding of end bar, 1.84 bits - 58%).

Taken together, these results suggest that the four temporal synergies of muscle activity correspond to four different phases of goal-directed voluntary movement. Thus, this specific temporal structure of muscle activity serves to convey complementary task information in time in order to characterize the movement evolution from the starting point to the endpoint.

### Functional role of spatial muscle synergies

Following the functional characterization of the temporal synergies, we performed a similar decoding analysis in the spatial dimension to investigate which task parameters are encoded by the activations of spatial synergies. We determined the functional role of each spatial synergy and then examined whether this function is consistent with the biomechanical function of the muscles each synergy activated.

Unlike temporal synergies that characterized all temporal task features in distinct movement phases, activations of each spatial synergy described a distinct subset of spatial task features in the entire movement duration. In particular, we identified spatial synergy activations that explain differences in the vertical dimension of movement (the first, second and fifth synergies shown in Fig. 3A) and others that explain differences in the horizontal dimension (the third and fourth synergies shown in Fig. 3A). Activations of the first spatial synergy discriminate mainly the height of the starting and end target (64% correct decoding and 0.56 bits - 36%, 62% correct decoding and 0.30 bits - 19% respectively- Fig. 3B,C, chance level for percent correct decoding is 33% and significance level is 36.5% for all 3-wise discriminations in this section). As this synergy activates muscles in the left (non-dominant) hemibody, it likely serves for maintaining posture and equilibrium when the subjects pointed to the respective targets. Starting height and the vertical direction of motion are also distinguished using the second spatial synergy (70% and 69% correct decoding; 0.63–40% and 0.52 bits - 33% respectively), while the fifth spatial synergy characterizes the vertical dimension of motion, i.e. starting and end heights and vertical direction (75%, 76% and 65% correct decoding, 0.68 bits - 43%, 0.73 bits - 46% and 0.40 bits - 25% respectively). The two latter synergies activate different sets of muscles in the right hemibody (primarily upper leg-trunk muscles and upper body-arm muscles respectively), thus they likely contribute complementarily to the production of motion in the vertical dimension. Recruitment of the third and fourth spatial synergies encodes the task parameters relating to the horizontal dimension of motion, i.e. starting bar (62% and 62% correct decoding, 0.38 bits - 24% and 0.32 bits - 20% respectively), horizontal direction (66% and 62% correct decoding, 0.49 bits - 31% and 0.39 bits - 25% respectively) and end bar (63% and 68% correct decoding, 0.34 bits - 21% and 0.35 bits - 25% respectively). The third spatial synergy activates mainly left lower leg and right lower body muscles and these activations discriminate bar B3 from the two other bars and the rightward direction of motion from the two other directions (see corresponding confusion matrices). Equivalently, the fourth spatial synergy comprises activations of right lower leg and left lower body muscles and discriminates bar B1 and the leftward direction of motion (see corresponding confusion matrices).

We also investigated how well the spatial synergies discriminated the temporal task parameters and vice-versa. This served as a direct comparison between temporal and spatial synergies in order to dissociate their respective functional roles. We found that considerably less spatial (temporal) task information was carried by temporal (spatial) synergies (for comparison, spatial synergies carried 0.79 to 1.19 bits about the starting target, 0.52 to 0.66 bits about the movement direction and 0.48 to 1.29 bits about the end target).

Overall, similarly to the temporal synergies, the spatial synergies serve distinct motor functions that can be explained in terms of their muscle composition. In contrast to the temporal synergies that relate to temporal task features, the structure and shape of the spatial synergies relate task parameters pertaining to the spatial dimension of motion (either vertical or horizontal) over the full time-course of movement. Importantly, when combining all spatial and temporal synergies, sufficient task information is conveyed to allow unequivocal characterization of the movement performed on each trial (for the example subject 85% correct decoding and 5.38 bits across the 72 pointing movements, i.e. 87% of the total information afforded by this set of movements which is 6.17 bits).

### Functionally similar muscle synergies across subjects

After characterizing the modular organization of muscle activity for the example subject, we examined whether the same structure holds for the muscle activity of all four participants we tested. Thus, we aimed to identify functionally equivalent spatial and temporal synergies across participants, i.e. synergies whose activations distinguished the same task parameters. To achieve this, we implemented a clustering procedure to group the extracted synergies of all participants based on the similarity of their movement decoding confusion matrices (see *Materials and Methods* - *Synergy selection and clustering* - *Clustering based on functional similarity* and Fig. 7 for details).

**Figure 4:**
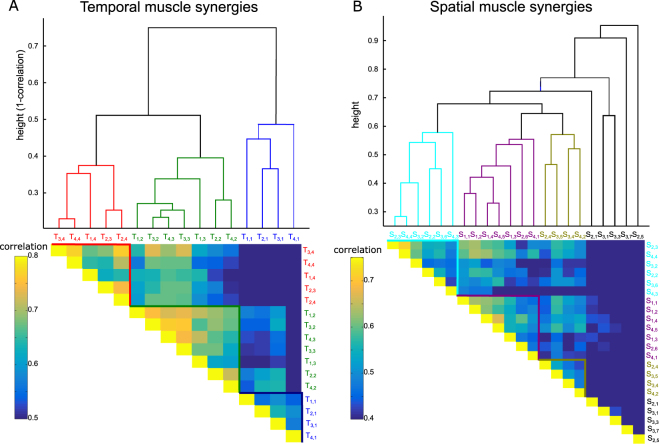
Hierarchical cluster trees and the corresponding functional similarity matrices for the temporal muscle synergies (**A**) and the spatial muscle synergies (**B**) across all subjects. Synergy T_*a*,*b*_ represents the *b*th temporal synergy (S for spatial synergies) of subject *a*. Color-coded branches of the hierarchical trees represent highly similar synergies that are clustered together. Synergies are clustered based on the correlation of their confusion matrices. Pairwise correlation of the confusion matrices of synergies *i* and *j* is shown as entry (*i*, *j*) of the functional similarity matrix (see color-coding of similarity matrix regions and synergy labels).

We found three clusters of temporal synergies and three clusters of spatial synergies. Figure 4A,B shows the hierarchical trees obtained from the hierarchical clustering algorithm as well as the functional similarity matrices for the temporal and spatial synergies respectively (clusters are color-coded). As can been seen in the similarity matrices, the clustering algorithm grouped synergies with high similarity (correlation between confusion matrix entries).

Although clustering was based solely on task decoding results, the three resulting temporal clusters (blue, green and red) contained synergies with similar activations in time (early, transient and late activations respectively), which indicates that the functional role of temporal synergies is robust and highly related to their temporal occurrence. Regarding the spatial synergies, three clusters (cyan, purple and yellow) were formed all containing synergies across different subjects. Our clustering analysis also specified a few spatial synergies that were functionally dissimilar to others (shown in black in Fig. 4) and thus were not included in the formed clusters. In the next section, we explain the function of these excluded synergies and also analyze the task coding role of each cluster.

### Task coding function of muscle synergy clusters

**Figure 5:**
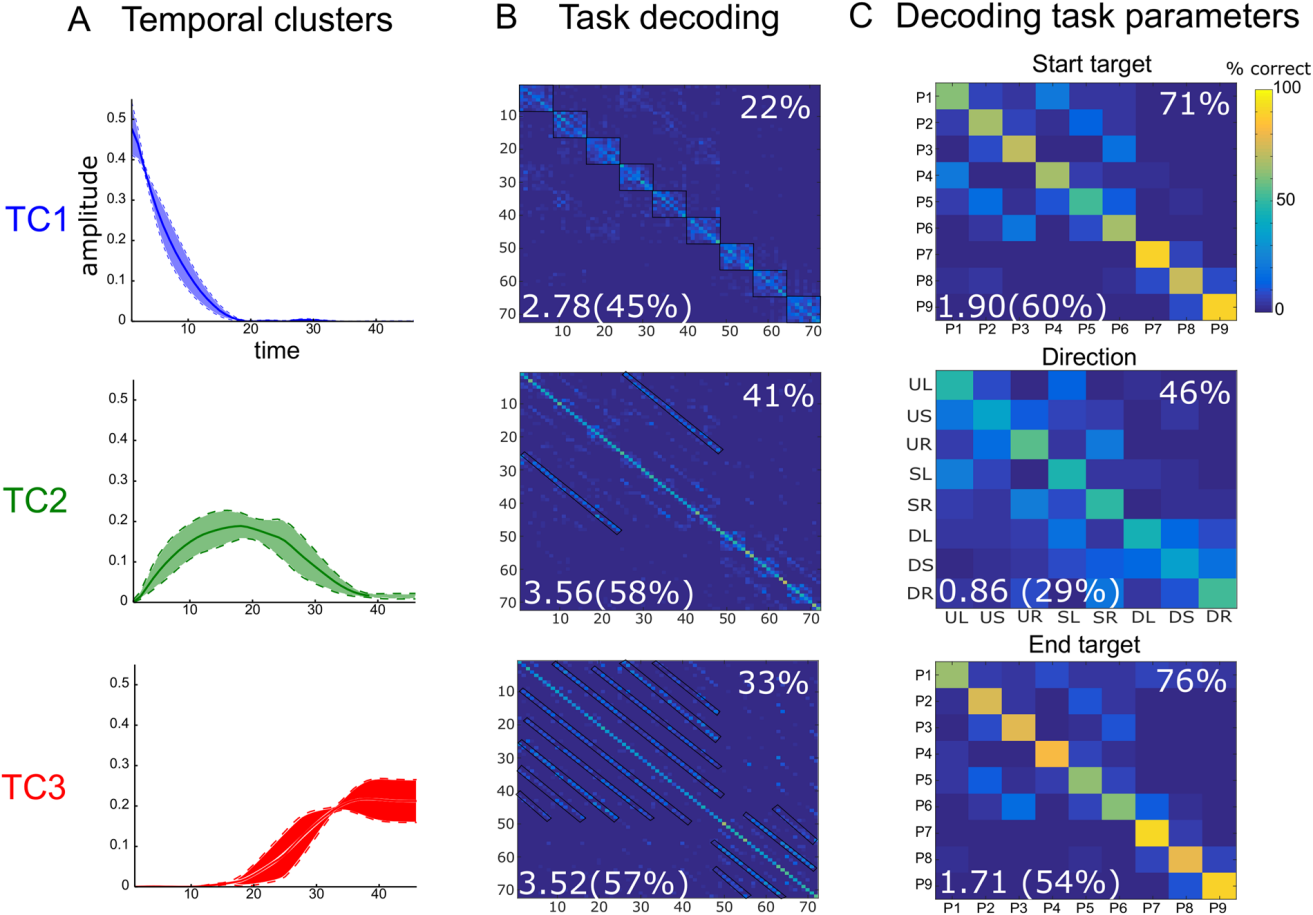
The three clusters of temporal muscle synergies (TC1, TC2, TC3 shown in rows) identified by our functional similarity clustering approach. (**A**) The average (±sem) profile of muscle activation of the temporal synergies belonging to each cluster. (**B**) The confusion matrix of all 72 movement directions (averaged across cluster members) for each cluster. (**C**) Confusion matrices of three task parameters describing fully three movement phases (beginning, transient phase and end of movement) that are encoded by the three clusters of temporal synergies (TC1, TC2 and TC3 respectively). Reported values on the bottom-left corner of each confusion matrix represent information values in bits and as percentages of the maximum information and values on the top-right corner represent percentages of correctly decoded trials (% correct). Matrix entries surrounded by black lines indicate common confusions.

We examined the composition of each cluster as well as its functional role in movement execution. The average temporal synergies (Fig. 5A) of the three clusters were consecutive in time, indicating that the identified clusters corresponded to three distinct temporal phases of movement. The first cluster (representing the early temporal synergies) discriminated the starting target of the movement (71 ± 5% correct decoding and 1.90 ± 0.15 bits - 60 ± 5% Fig. 5C-top). As shown in the confusion matrix of all 72 movements (Fig. 5B-top, corresponding colorbar is top-right), more confusions were found amongst movements having the same starting target and different end targets (corresponding values are organized in the confusion matrix as nine 8 × 8 squares around the diagonal indicated in black; 7% pairwise confusions on average). This confirms the ability of the first temporal cluster to distinguish better the beginning than the remaining part of the movement (38 ± 4% correct decoding of direction and 0.62 ± 0.07 bits - 21 ± 2%, 22 ± 2% correct decoding of end target and 0.13 ± 0.03 bits - 4 ± 1%). The second cluster (representing the transient temporal synergies) decoded the movement direction (46 ± 4% correct decoding and 0.86 ± 0.09 bits - 29 ± 3%, Fig. 5C-middle) but also the starting and end target (44 ± 4% and 45 ± 6% correct decoding - 1.16 ± 0.19 bits - 37 ± 6% and 0.89 ± 0.13 bits - 28 ± 4% respectively). The corresponding confusion matrix of all movements (Fig. 5B-middle) shows confusions between movements that have the same horizontal direction and endpoint and their starting locations differ only in the height dimension (see the lower and upper sub-diagonals of the confusion matrix indicated in black, 12% confusions on average). In particular, starting points T1, T2 and T3 (higher level) were confused as T4, T5, and T6 (middle level) respectively and vice-versa (see Fig. 1 for target positions).. The third temporal cluster (representing the late temporal synergies) characterized the movement end target (76 ± 7% correct decoding and 1.71 ± 0.07 bits - 54 ± 2% vs. 23 ± 3% correct decoding and 0.39 ± 0.03 bits - 12 ± 1% for starting target and 40 ± 5% correct decoding and 0.82 ± 0.04 bits - 28 ± 1% for direction, Fig. 5C-bottom). This cluster confused movements having different starting points and directions but the same end target, as illustrated by the black lines parallel to the diagonal on the confusion matrix (Fig. 5B-bottom, 8% confusions on average). In general, bottom targets were consistently distinguished from the other two target heights probably because of the higher involvement of lower body muscles required for movements from/to bottom targets.

We also note that, although percent correct decoding of all 72 movements by each temporal cluster is not very high (22 ± 5% for TC1, 41 ± 3% for TC2, 33 ± 2% for TC3), a high amount of task information is carried by the three clusters (2.78 ± 0.24 for TC1, 3.56 ± 0.17 for TC2, 3.52 ± 0.06 for TC3 corresponding to 45 ± 4%, 58 ± 3% and 57 ± 1% of the total task information respectively). This finding derives from the fact that confusions are stereotyped and clustered depending on the cluster’s functionality (as indicated by the black areas in the confusion matrices in Fig. 5), which translates into higher task information (see *Materials and Methods* - *Quantifying task information* for details).

**Figure 6:**
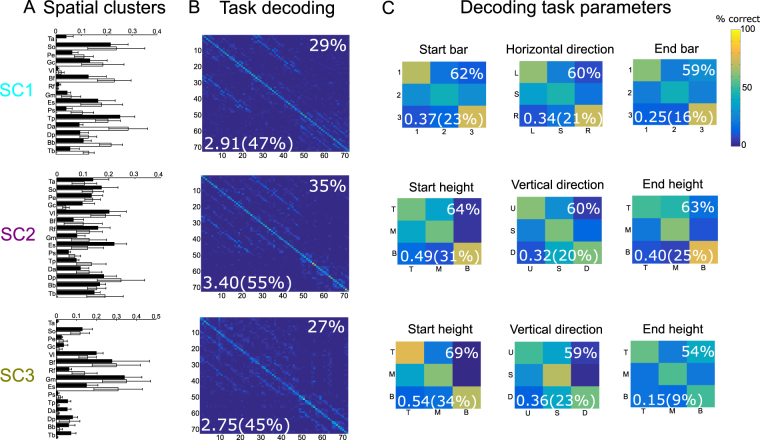
The three clusters of spatial muscle synergies (SC1, SC2, SC3 shown in rows) identified by our functional similarity clustering approach. (**A**) The average (±sem) muscle activation of the spatial synergies belonging to each cluster. (**B**) The confusion matrix of all 72 movement directions (averaged across cluster members) for each cluster. (**C**) Confusion matrices of six task parameters describing the spatial location of the subject’s hand over the entire movement duration: starting bar, horizontal direction and end bar (encoded by SC1) describing the horizontal dimension of motion and starting height, vertical direction and end height (encoded by SC2 and SC3) describing the vertical dimension of motion. Reported values on the bottom-left corner of each confusion matrix represent information values and on the top-right corner percentages of correctly decoded trials (% correct).

With regard to spatial synergy clustering, the three clusters consisted of muscle activations in the whole body (Fig. 6A, top three panels). The first cluster activated mainly a) leg and lower body muscles that serve for side-to-side rotation and b) upper body and arm muscles that serve for arm movement (Fig. 6A-top). Consistent with the muscles’ function, this spatial cluster contributed to the horizontal dimension of the movement, i.e. starting bar, horizontal direction and end bar (62 ± 5%, 60 ± 4% and 59 ± 5% correct decoding; 0.37 ± 0.08 bits - 23 ± 5%, 0.34 ± 0.08 bits - 21 ± 5% and 0.25 ± 0.04 bits - 16 ± 3% respectively, Fig. 6B,C-top). In support of this claim, bar B3 on the participant’s left, which involves the largest body rotation, was decoded more accurately than the other two bars (73% on average), whereas bar B2 in front of the participant, which requires the smallest body rotation, was decoded incorrectly more often (43% on average). Regarding the second cluster, its average muscle activations spanned the whole body almost uniformly (Fig. 6A-middle). This cluster mainly served to decode the vertical direction of movement, i.e. starting height, horizontal direction and end height (64 ± 3%, 60 ± 1% and 63 ± 4% correct decoding - 0.49 ± 0.06 bits - 31 ± 4%, 0.32 ± 0.04 bits - 20 ± 3% and 0.40 ± 0.08 bits - 25 ± 5% respectively, Fig. 6B,C, middle). We found higher discriminability for the bottom targets (75% on average), whereas the other two heights were more often confused with each other (36% pairwise confusion on average). These confusions were partly resolved by the the third cluster that comprised activations of upper leg and trunk muscles (Fig. 6A, third row from top) and discriminated the top starting targets from the middle ones (77% correct decoding of top starting targets, 69 ± 3% correct decoding of starting height in general, 0.54 ± 0.07 bits - 34 ± 4%) and same height movements from the upward movement direction (70% correct decoding of same height movements, 59 ± 3% correct decoding of vertical direction in general, 0.36 ± 0.08 bits - 23 ± 5%, Fig. 6B,C-bottom).

Our clustering analysis also identified 5 spatial synergies (from the two subjects having larger sets of spatial synergies) that bore no functional similarity to any other. When investigating the task relevance of these synergies, we found that they related poorly to the task at hand (1.37 ± 0.33 bits, i.e. 22 ± 5% of the total task information - 13 ± 3% correct decoding). and did not exhibit a strong relation to any of the considered task parameters (<0.15 bits and <50% correct decoding for all 3-wise discriminations). We then examined the muscle composition of these spatial synergies to gain more insights about the biomechanical function of the muscles they comprise. We observed that they activated primarily a) upper body and arm muscles that serve for raising or lowering the right arm, which is a common feature of all pointing movements considered here and b) activations of muscles in the left hemibody that probably do not pertain to phasic muscle activity for task accomplishment but rather tonic activations shared across movement directions for maintaining posture and equilibrium. Activations of these subject-specific synergies, though not informative about the task, are useful for the construction of genuine muscle patterns in order to perform this set of movements.

In sum, our task-level analysis uncovered three temporal and three spatial muscle synergies that summarize the composition and the functionality of individual participants’ synergies. These temporal (spatial) synergies are consistently formed across subjects in order to describe the temporal (spatial) features of the task and, when taken together, they characterize the motor task at hand.

## Discussion

In this study, we linked temporal and spatial muscle synergies to task parameters and derived a task representation intepretation of modular muscle activity in a whole-body pointing task. Our approach relied on the design of a motor task comprising a large number of distinct movements (72) coupled with the use of a computational scheme for the single-trial analysis of synergy activations. This methodology allowed us to assess how individual muscle synergies relate to the main features of the task at hand. Our findings indicate that motor signals, as inferred from EMG data, have a low-dimensional structure based on the combined activations of a) temporal synergies that encode distinct movement phases (initiation, transient and termination) and b) spatial synergies that encode distinct 3-dimensional spatial directions between initial and final positions. Crucially, both types of synergies capture complementary, not redundant nor irrelevant, aspects of the task that are essential for the characterization of the performed movement over time and space. Finally, by introducing a novel functional similarity analysis, we identified a small set of temporal and spatial synergies that, despite differences in their activation profiles, had synergistic functional roles in movement execution consistently amongst participants. The significance of these results is discussed in what follows.

### From task parameters to muscle activations and vice-versa

Our findings are compatible with a hierarchical organization of motor signals for the production of muscle patterns that are effective in task space, as suggested in several works^18,33–36^. The analyses we proposed here describe a direct link between the task and muscle levels of such a hierarchical scheme^15,37,38^. In this framework, the CNS converts task-level movement goals/parameters to activation coefficients in synergy space during movement planning. During movement execution the reverse mapping is performed by the CNS, i.e. synergy recruitment gives rise to the construction of genuine muscle patterns adequate to accomplish the task at hand^39^. Hence, spatial and temporal muscle synergies may represent the pivot of such a hierarchical neural control structure in which higher-level brain circuits operate on task-related variables^8,40^ and lower-level circuits construct full muscle activities by combining descending motor commands with reflex contributions to effectively produce movements^10,41^.

Consistent with this framework, electrophysiological and neuroimaging evidence has demonstrated that higher levels of the nervous system encode abstract motor representations^38,42,43^. In particular, in line with our findings, higher-order motor areas have been shown to carry independent yet interacting representations of the spatial and temporal movement features. These representations may serve as a modular feature-separating storage that allows flexible recombination and efficient encoding of complex motor behaviors^44,45^. Then, the output of the cortical hierarchy, i.e. the primary motor cortex, has been shown to coordinate relevant actions^46–50^. At a lower level of the motor system, neural circuits in the brain stem and spinal cord have been suggested as a neural basis for coding the modules that are activated by descending commands from supraspinal areas possibly combined with sensory impulses from afferent pathways^51–57^. Finally, at the muscle activation level, motor unit decomposition of EMG activity has shown that synergistically activated muscles are controlled primarily by a shared neural drive^58^.

We also note that the modular organization identified in the present complex whole-body movements likely differs from physiological modules that are hardwired in the spinal systems and activate distinct limb effectors^7,59–61^. Here, the spatial and temporal synergies describe coordinated muscle activity across multiple limb effectors and body parts, thus they might represent tightly managed cross-effector coordination of more basic spinal motor modules. The coupling of several muscles across limbs as shown in the spatial synergies and the temporal succession of muscle activations indicated by the temporal synergies might be a motor structure that the CNS implements in order to map the fundamental modules onto motor goals and reduce the dimensionality of the control problem.

### Task-relevance of extracted temporal and spatial muscle synergies

The novelty of our results lies in the characterization of the extracted temporal and spatial synergies in terms of the movement features they account for. We found that certain task parameters could be discriminated by means of a single spatial or temporal synergy (or few synergies), unlike the usual approach that takes into account the ensemble activation of all synergies to deduce task characteristics such as motion direction^22,62,63^. Crucially, this shows that some task parameters are not only described by complete synergy combinations but may be coded by the synergy itself. In particular, the distribution of activation patterns across muscles (spatial modularity) was shown to map to the motion spatial features (e.g. upward vs. downward and leftward vs. rightward directions), which is compatible with the directional tuning observed in previous studies^16,63–66^. Similarly, the distribution of activation patterns in time (temporal modularity) was shown to map to the motion phases suggesting that successive motor commands are generated and sent to adequate groups of muscles to transition from a starting point to an end point^38,44,67–71^.

Thus, our findings may be taken as evidence that space-by-time modularity constitutes a meaningful representation of muscle activity that carries the main temporal and spatial information of the task in a principled and concise way as follows. Temporal synergies segment the course of movement so as to convey the crucial information about the initial/final postures and the transient impulses to accelerate or decelerate the body towards the target. Temporal synergies with an early burst are distributed to the spatial synergies necessary for maintaining posture, followed by other temporal synergies relevant for the transient movement features (horizontal/vertical directions), and temporal synergies with later bursts specify more finely the characteristics of the motor goal such as the final posture^72,73^. In space, modular motor signals are distributed across muscles in the entire body to serve functions varying from static prerequisites, such as maintaining equilibrium at an initial or final posture, to dynamic prerequisites, such as accelerating the body towards some spatial location in order to reach a desired target^62,64,74^. This temporal and spatial organization potentially provides a sophisticated modular motor signaling system that is at least effective in the characterization of essential task features. We speculate that this modular structure may be the outcome of developmental or learning processes^75^ that transform habitual motor patterns into temporal and spatial motor building blocks that conform to the structure of the underlying movements.

### Clusters of functional muscle synergies

A corollary of the current study is the proposal of a “functional similarity” approach aiming to cluster synergies from different subjects that have similar functional roles as represented by the task parameters they encode. A similar approach, termed representational similarity analysis (RSA), has been proposed for quantifying similarity between representations of visual objects using brain imaging^76,77^. In the EMG dataset we analyzed here, similarity of the activation profiles of the extracted synergies was very high for the temporal synergies across subjects (0.92 average correlation) but relatively low for the spatial synergies (0.52) which may be explained by physical discrepancies (muscle sizes, skin conductance etc.) and kinematic differences between participants or as an outcome of human development as different synergies may be learned by different individuals depending on their motor preferences^78^. Also, across-subject variability in the reflex-mediated contributions to EMGs may have played a role in the different number of spatial synergies^56^. More generally, muscle synergy variability across individuals has been a strong concern in behavioral neuroscience and neuro-rehabilitation^79–82^. This observation motivated the necessity of new methodologies to assess synergy similarity across humans and enhance personalization of treatments. We observed that although the extracted spatial synergies may be different in shape (low activation similarity), they may have similar functional roles^27,83^. Thus, we proposed computing the “functional similarity” of the synergies, i.e. similarity between their confusion matrices, which in effect uncovered a few clusters of synergies that share the same task coding function despite shape differences. This novel approach opens up new perspectives for matching synergies of different individuals, which may find useful applications in therapeutic and rehabilitation research.

In practice here, for the temporal synergies, the identified functional similarity clusters appear to correspond well to temporal activation clusters with the additional advantage that functional similarity clustering indicated that the two middle/transient temporal synergies should be merged into a single cluster. Such a finding cannot be obtained if we cluster synergies based on activation similarity, which groups the two transient synergies into separate clusters because they have different activation timings (yielding 4 temporal custers in total). The identified 3-dimensional representation of muscle activations in time is also consistent with previous studies demonstrating a tri-phasic pattern of muscle activity during voluntary motion varying from single-joint to whole-body movements^84–86^. With respect to the spatial synergies, the low activation similarity we noted above is also observed in the high variability of the average muscle activations of the three spatial clusters (see error bars in Fig. 6A), indicating that spatial synergies belonging to the same cluster may have different muscle activation ratios. Nevertheless, these synergies share the same functional role for all individuals performing this motor task. We also note that if we cluster spatial synergies based on their activation similarity (see^32^ for an illustration of the spatial synergies of all subjects), we obtain 7 or more clusters (depending on the cutoff threshold), a much higher dimensionality than the one obtained with the proposed functional clustering.

### Limitations and future work

A limitation of the proposed approach is its applicability to the decoding of discrete task parameters only. In fact, decoding the full time course of kinematic or kinetic signals from synergy activations, which entails extending our decoding method to deal with continuous task variables, would provide further support for the effectiveness of the modular decomposition. However, in this study the time courses of kinematic and kinetic signals as well as the movement speed constitute motor choices made by the participants. Hence, we preferred to restrict our analysis to task parameters that were imposed to the participants by the experimental protocol and were thus identical across them such as start/end targets and overall motion directions. Nevertheless, the joint analysis of muscle activations and kinematic or kinetic data constitutes an interesting direction for future research. Concerning movement speed, results from previous work on planar arm reaching movements showed that varying the speed instructions (i.e. including different speed conditions) did not alter the structure of the identified modular decomposition or the task decoding scores^16^. To test this here, we applied the space-by-time decomposition to EMG datasets containing a) only the slowest trial and b) only the fastest trial of the example subject (E4) for each task. The extracted synergies showed significant similarity to the synergies extracted from the full dataset of E4 and to each other – average similarity between the two sets of synergies (high vs. low speed) was $\bar{r}=0.99$ for the temporal synergies and $\bar{r}=0.83$ for the spatial synergies (all *ps* < 0.01). Although more experimental work is required to test this, movement speed variations are expected to yield variations in muscle activity levels and consequently in the activation coefficients but would not affect the task coding role of each muscle synergy. In particular, we predict that results regarding initial and final postures would remain unaltered as they mainly relate to tonic muscle activities, but direction is likely to be harder to decode as speed decreases due to the lower signal-to-noise ratio of EMG signals at lower movement speeds.

A second limitation is that the method does not account for all the variance in the recorded EMG data. The pre-processing of the signals and the approximate nature of the decomposition ineluctably leads to some loss of information. Reducing this loss would require including higher frequency variations in the modular representations. Here, the low-pass filtering combined with the subsampling of the EMG waveforms likely limits the temporal/phase resolution of the temporal decomposition even though more temporal synergies could be extracted to get a finer temporal resolution. Also, currently the proposed temporal decomposition assumes fixed temporal synergies with no temporal or phase variability parameters. To assess the effect of these limitations of our approach on the signal approximation and task discrimination quality, we repeated our analysis a) after increasing the low-pass threshold to 5, 10 and 20 Hz and b) on trial-averaged EMG data using a space-by-time decomposition that includes time shifts for the temporal synergies. We found that the decoding performance of the decompositions obtained when using higher low-pass filters did not change significantly whereas the VAF gradually decreased with higher low-pass filtering thresholds and increased when time shifts were included. This indicates that these filtered temporal variations did not contribute significantly to the description of task differences although they were useful for the better approximation of the EMG recordings. However, capturing higher frequency variability may be necessary for the decoding of the full time course of movement trajectories or finer parameters of the underlying movements. In fact, it has been shown that the phase of temporal synergies contributes to trajectory control in vertebrates^71,87^ as well as human balance control^88,89^.

In sum, this study introduces a computational approach for the functional characterization of muscle synergies derived from EMG data, which may be a useful tool for the analysis of muscle synergy decompositions in task space. Although existing modularity models and the relevant analysis techniques provide a descriptive account of muscle activity and its relation to the task at hand, we suggest that further theoretical and experimental work will be required to a) assess the potential neural basis of modularity and b) unify the modularity hypothesis with other motor control theories such as the optimality and the equilibrium point hypotheses^8,13,33,90–95^.

## Materials and Methods

### Experimental procedure

#### Subjects

Four healthy right-handed participants (E1, E2, E3, E4, 2 males, aged = 25 ± 3 old, height = 1.72 ± 0.08 m, weight = 70 ± 7 kg) with no history of neuromuscular disease voluntarily participated in the experiment. The experimental protocol was approved by the Dijon Regional Ethics Committee and conformed to the Declaration of Helsinki. Written informed consent was obtained by the subjects following guidelines of the Université de Bourgogne.

#### Task

Participants executed whole-body point-to-point movements in various directions at a self-selected pace. In brief, the experimental protocol (detailed in^32^, and illustrated in Fig. 1) specified 9 targets on 3 vertical bars. Each bar had 3 targets on different heights determined based on the participant’s height. Participants stood barefooted and performed pointing movements between all pairs of targets (i.e. a total of 72 different pointing movements or “tasks”) using the index fingertip of their dominant right arm. The left arm as well as the rest of the body were not constrained.

As our protocol specified a large number of experimental conditions and we also wished to employ a single-trial analysis, we aimed to record a large number of trials for each participant. Thus, we asked each participant to perform the experiment in two separate sessions in consecutive days. On each session, subjects performed 15 repetitions of each movement (30 repetitions in total), which resulted in a total of 72 × 30 = 2160 recorded trials per subject. Our aim here was to obtain high statistical power for our single-trial analysis, for this reason we chose to collect more data from a relatively small number of subjects (pooling trials from two sessions) rather than have more participants perform fewer trials. Nevertheless, when considering the EMG data of each recording session separately (8 datasets in total), we obtained qualitatively very similar findings, thus hereafter we will present the results obtained from the analysis performed on the full EMG data of each subject (pooled across sessions). Given the small number of participants, we will not perform statistical comparisons at the population level, instead we will report results for individual subjects.

#### EMG recording and preprocessing

We recorded the activity of 30 muscles by means of an Aurion (Milan, Italy) wireless surface EMG system. The skin was shaved before electrode placement, and abraded softly. EMG electrodes were placed symmetrically on the two sides of the body on the following muscles: tibialis anterior (Ta), soleus (So), peroneus (Pe), gastrocnemius (Ga), vastus lateralis (Vl), rectus femoris (Rf), biceps femoris (Bf), gluteus maximus (Gm), erector spinae (Es), pectoralis superior (Ps), trapezius (Tp), anterior deltoid (Da), posterior deltoid (Dp), biceps brachii (Bb), triceps brachii (Tb). Before the experiment, participants were asked to perform isometric muscle contractions while EMG signals were monitored in order to optimize recording quality and ensure that EMG recordings were not contaminated by cross-talk from neighboring muscles. Reliability of recordings and possible detachments of EMG sensors were assessed by visually checking the signal amplitude of the recorded muscles to identify abnormal changes across trials. These movement artifacts were visually removed by discarding the associated trials (<2% of the total number of trials). We simultaneously recorded the 3D position of a retroreflective marker (diameter = 20 mm) placed on the right index fingertip of the subjects using an optoelectronic measuring device (Vicon Motion System, Oxford, UK) at a sampling frequency of 100 Hz. The recorded finger kinematics were low-pass filtered (Butterworth filter, cut-off frequency of 20 Hz) and numerically differentiated to compute tangential velocity. We defined movement onset (*t*_0_) and end (*t*_*end*_) times as the times between which the fingertip velocity superseded 5% of its maximum and restricted our analysis to the interval (*t*_0_ − 100 ms, *t*_*end*_) of EMG activity^22^. These movement-related EMGs for each trial were first high- pass filtered (20 Hz) and then digitally full-wave rectified and low-pass filtered (Butterworth filter, cut-off frequency of 3 Hz, zero-phase distortion^96^) and normalized to 1,000 time steps. A final waveform of 50 time steps was then obtained by using trapezoidal integration of the latter signal on a uniform temporal grid. The EMG signal of each muscle was then normalized in amplitude by dividing each single-trial muscle signal by its maximal value attained throughout the experiment. For each subject, we finally formed an EMG matrix of (50 time steps × 30 muscles) in rows and 2160 trials in columns consisting of all the movement-related EMG activity (rectified and filtered) of the 30 muscles for all recorded trials. This matrix was used as input to the modular decomposition algorithm to characterize the spatial and temporal structure of muscle activations for this set of movements. Figure 1B shows both raw and filtered EMG signals for three movement directions: P1–P3 (leftward movement from top right to top left), P1–P7 (downward movement from top to bottom on the right side), and P1–P9 (diagonal movement from top right to bottom left).

### Space-by-time modular decomposition of muscle activity

#### Space-by-time decomposition model

We used a tensor decomposition^97,98^ with non-negative constraints^16,99^ to decompose the single-trial EMG signals into spatial and temporal synergies. This modularity model^16^ represents muscle activity as a linear combination of separate but concurrent spatial and temporal synergies combined in single trials by scalar coefficients.

According to the space-by-time factorization, a single-trial muscle pattern ${{\rm{M}}}^{l}\in {{\mathbb{R}}}_{+}^{T\times M}$ can be written as a three-factor multiplication (*T* and *M* being the number of time frames and muscles, respectively):

1 $${M}^{l}\approx {W}_{t}{A}^{l}{W}_{s}\,\forall \,l\in [1,L]$$

where ${W}_{t}\in {{\mathbb{R}}}_{+}^{T\times K}$ is a matrix whose columns are the temporal synergies, ${W}_{s}\in {{\mathbb{R}}}_{+}^{N\times M}$ is a matrix whose rows are the spatial synergies and the matrix $${A}^{l}={({a}_{ij}^{l})}_{\begin{array}{l}1\le i\le K\\ 1\le j\le N\end{array}}$$ includes all single-trial activation coefficients. The parameters *K* and *N* correspond to the number of temporal and spatial synergies respectively and are free parameters of the decomposition model. Note that the matrices *W*_*t*_ and *W*_*s*_ are inferred from all trials and thus are shared across trials (i.e. independent of any particular trial), and constitute the invariant temporal and spatial synergies and *A*^*l*^ are the trial-dependent activation coefficients combining the synergies in order to perform each individual movement.

#### Variance accounted for (VAF)

To assess how well the space-by-time decomposition reconstructed the original EMG recordings, we computed the Variance Accounted For (VAF) by the decomposition^62^. VAF is a measure of goodness of fit and is defined as the total approximation error divided by the total variance of the dataset:

2 $${\rm{VAF}}=1-\sum _{l}\,\parallel {M}^{l}-{W}_{t}{A}^{l}{W}_{s}{\parallel }^{2}/\sum _{l}\,\parallel {M}^{l}-\bar{{\bf{m}}}{\parallel }^{2},$$

The total approximation error is computed as the squared Frobenius norm (||.||) of the difference between the original muscle activity and its approximation by the space-by-time decomposition and the total variance of the dataset is the squared Frobenius norm of the difference between the original muscle activity and the mean EMG activation across all trials ($\bar{{\bf{m}}}$).

#### Synergy extraction

To identify spatial synergies, temporal synergies and single-trial activation coefficients from the recorded muscle activity, we applied sNM3F, a NMF-based synergy extraction algorithm. sNM3F implements the space-by-time decomposition, i.e. identifies spatial and temporal components as well as activation coefficients that describe the performed movements^16^. The advantage of NMF-based decompositions over other dimensionality reduction methods (e.g. PCA, ICA) is that they restrict the extracted synergies and activations to be non-negative, which makes them physiologically relevant for EMG signals as muscles cannot be activated “negatively”. We also used a supervised version of this algorithm incorporating task constraints (i.e. labelling each trial with the task (1, …, 72) that was performed during the trial) in the synergy extraction method (named DsNM3F^17^) and found qualitatively very similar results. Thus, in the following we present and analyze only the synergies identified by sNM3F.

We input the preprocessed EMG matrix (see above) of each subject to sNM3F and extracted *K* temporal synergies, *N* spatial synergies and *K* × *N* × *L* activation coefficients to describe the EMG activity underlying execution of the movements under consideration. The numbers of spatial and temporal synergies (*K* and *N* respectively) are free parameters of the algorithm, thus we varied *K* = 1, …, 10 and *N* = 1, …, 10 and computed the decomposition for all the 100 possible (*K*, *N*) pairs. To overcome convergence to local minima, for each (*K*, *N*) we ran the algorithm 50 times using random initializations of the parameters and selected the output that gave the lowest reconstruction error. We then used a decoding analysis to determine the smallest set of synergies capturing all task and trial variations of the data (see *Synergy selection and clustering*).

### Decoding analyses

#### Task parameters

Our experimental design specified 9 targets placed on 3 different heights of 3 vertical bars (see 0 for an illustration), which defined 72 distinct pointing movements. Each of these movements can be fully described by the starting target (P1, …, P9) and end target (P1, …, P9) of the corresponding movement. Alternatively, each movement can be fully characterized by the bar on which the starting target lies (1, 2, 3) together with the height on which the target is placed (top - T, middle - M, bottom - B) and the corresponding bar (1, 2, 3) and height (T, M, B) of the end target. Other parameters describing features of the performed movement are a) the vertical direction and b) the horizontal direction of the movement, i.e. a) whether the subject had to move towards the left (L), towards the right (R) or stay on the same bar (S) and b) whether they had to move upwards (U), downwards (D) or stay at the same height (S). Taken together these parameters define the movement direction in the 3-dimensional task space which takes 8 distinct values: up-left (UL), up-same (US), up-right (UR), same-left (SL), same-right (SR), down-left (DL), down-same (DS), down-right (DR). In sum, to describe task differences, we used the following set of task parameters: starting point, starting bar, starting height, direction, horizontal direction, vertical direction, end bar, end height, end target, and finally the full movement direction (1, …, 72).

To characterize separately the temporal and spatial dimensions of the task at hand, we defined two groupings of the task parameters. The first grouping carried the temporal information of the task and consisted of parameters describing: a) the beginning of the movement (starting target, bar and height), b) the transient movement phase (direction, horizontal direction and vertical direction) and c) the movement end (end target, bar and height). The second grouping carried the spatial information of the task and consisted of parameters describing: a) the horizontal dimension of movement (starting bar, horizontal direction, end bar) and b) the vertical dimension of movement (starting height, vertical direction, end height) independently of the timing. We then aimed to assess which of these task parameters may be encoded by the single-trial synergy activations and which synergies carry information about the temporal and the spatial task features. To do this, we quantified how reliably recruitment of each synergy can be mapped onto each task-related parameter as described below.

#### Decoding task parameters

The single-trial activation coefficients of the space-by-time decomposition may represent the motor commands that recruit spatial and temporal synergies and encode their level of activation in individual trials. Specifically, the activation coefficient ${a}_{i,j}^{l}$ represents the relative amplitude of temporal synergy *i* in the muscles defined by spatial component *j* on trial *l*. If a particular temporal/spatial synergy exhibits different activation strengths depending on a particular task parameter, these differences will be reflected on the values of the activation coefficients *A*^*L*^. Thus, the activation coefficients can be used as the single-trial parameters that relate each synergy to the movement performed in each trial or any of the task parameters characterizing each trial^100^. Hence, to test if the space-by-time synergy recruitment allows decoding task parameters, we employed a single-trial classification analysis that used as parameters the activation coefficients *A*^*L*^. In particular, we used a linear discriminant analysis (LDA) in conjunction with a leave-one-out cross-validation and quantified decoding performance as the percentage of correctly decoded trials^22^.

To characterize the functional role of each synergy, we predicted the task parameter values on each trial from the synergy activations. To compute the decoding performance of each temporal (spatial) synergy, we used as input to LDA the *N* (*K*) activation coefficients combining it on each trial with the *N* spatial (*K* temporal) synergies. These analyses allowed us to a) tease apart the contribution of each temporal/spatial synergy to the decoding of each task parameter, b) determine the synergies that account for the temporal and the spatial aspects of the task and c) evaluate whether the assumed space-by-time modularity is compatible with unequivocal task characterization.

#### Confusion matrices

To illustrate which task parameters are discriminated reliably and which are typically confused, we reported decoding results in the form of confusion matrices^21^. The values on a given row *i* and column *j* of a confusion matrix *D*(*i*, *j*) report the fraction of trials in which we decoded a value *j* of a task parameter while the actual value of this parameter in that trial was *i*. Hence, the confusion matrices illustrate not only the percentage of correctly decoded trials but also the distribution of decoding errors, thereby showing which combination of parameters tend to be confounded. To gain more insights about the functional role of the spatial and temporal synergies with respect to task performance, we plotted separate confusion matrices for all synergies and task parameters. Unless otherwise stated, for each synergy we present confusion matrices of the task parameters that it discriminated best.

#### Quantifying task information

Synergy activations may carry information about the task at hand by means other than just reporting the most likely value of each task parameter (quantified by % correct decoding). For example, given the activation of a specific synergy, some values of a task parameter may be utterly unlikely and others very likely^21^. Thus, to include these aspects of task information in our assessment of the task coding function of each synergy, we computed for each task parameter and synergy the mutual information *I*(*T*; *T* ^*p*^)^101^ between the rows and columns of the corresponding confusion matrix. In other words, *I*(*T*;*T* ^*p*^) is the mutual information between the actual value of the task parameter and the one predicted by the decoding algorithm given the synergy activations and is defined as follows:

3 $$I(T;{T}^{p})=\sum _{t,{t}^{p}}\,P(t,{t}^{p})\,{\mathrm{log}}_{2}\,\frac{P(t,{t}^{p})}{P(t)P({t}^{p})}$$

where *t* is the actual value of the task parameter, *t*^*p*^ is the value predicted by the decoding algorithm, *P*(*t*) is the probability of task parameter *t*, *P*(*t*, *t*^*p*^) is the confusion matrix, i.e., the joint probability of predicting *t*^*p*^ when the actual value is *t*, and *P*(*t*^*p*^) is the probability of predicting *t*^*p*^ across all values of the task parameter. Information is measured in bits. Every bit of information reduces the overall uncertainty about the task by a factor of two. Perfect knowledge about the task parameter from the synergy activations gives maximum mutual information of *log*_2_ *V*, where *V* is the number of distinct values that the task parameter takes. Thus, we report information values both in bits and as percentages of the maximum information for each task parameter. We computed information values using the Information Breakdown Toolbox^102^. To eliminate the upward bias of information measures due to limited sampling, we used the Panzeri-Treves (PT) bias-correction method^103,104^.

Importantly, as noted above, this information measure is sensitive not only to the percentage of correctly decoded trials but also to the distribution of errors in the decoder, i.e., how task parameter values are confused. For a given % correct value, more information can be obtained if incorrect predictions are concentrated into clusters around the correct stimulus rather than distributed randomly^105,106^.

### Synergy selection and clustering

#### Selecting the number of synergies

We employed the decoding analysis described above to identify the most compact and task-discriminating space-by-time decomposition. We increased gradually the numbers of temporal and spatial synergies extracted (*K*, *N* respectively) and decoded the motor task performed on each trial for each *N* × *K*-dimensional decomposition. The optimal number of synergies was then selected as the step at which adding any supplementary synergy did not give any significant gain in task decoding performance (*p* > 0.05). To assess the significance of decoding performance, we employed a permutation test where we randomly shuffled the coefficients corresponding to the added component (while the distributions of all other coefficients were unaffected) and computed decoding performance [^22,23^, for more details].

#### Clustering synergies based on their functional similarity

We propose a novel method for clustering synergies across participants based on their functional similarity as revealed by the task decoding analysis. Typically in motor modularity studies, synergies are clustered using as criterion their activation similarity, i.e. temporal (spatial) synergies belong to the same cluster if they have similar temporal activation profiles (muscle activations)^16,22,29,69,107^. However, several factors, including individual idiosyncracies, motor choices and physiological differences in synergy formation, may lead to different muscle groupings and/or temporal activations, whereas the underlying synergies may have the same functionality with respect to task performance. Potential causes of these differences are physical discrepancies amongst participants (different muscle sizes, skin conductance etc.) as well as diversity in movement kinematics. Furthermore, the idenitified synergies may be the output of the recruitment of different (though motor equivalent) assemblies of fundamental physiological motor units that drive cross-limb actions. In such case, some of the extracted synergies may have dissimilar muscle activity compositions but similar functional roles in terms of the effective accomplishment of the task. Hence, here we propose clustering synergies of the same type (spatial or temporal) across participants based on whether they allow decoding the same task features. The core idea of this approach is that although synergies may be different in shape (low activation similarity), they should be grouped together if they decode the same task parameter.

**Figure 7:**
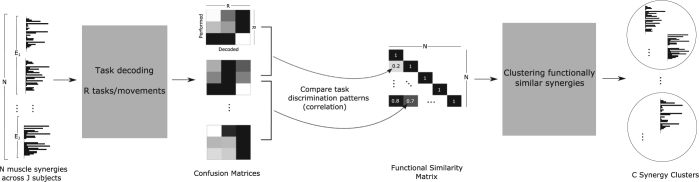
Schematic illustration of our computational approach comprising three steps: decoding, functional similarity analysis and clustering. We input the single-trial activations of each of the *N* muscle synergies to a linear decoding algorithm that predicts which of the *R* movements was performed on each trial We plot the decoding results in the form of *R* × *R* confusion matrices. Each entry of the confusion matrix *D*(*i*, *j*) represents the fraction of trials on which movement *i* was decoded as *j*. Then we compare the confusion matrices of all synergies by computing their pairwise correlations and summarizing them in a *N* × *N* lower triangular functional similarity matrix. Finally, we use a hierarchical clustering algorithm to group functionally similar synergies onto *C* distinct clusters.

In the following, we present the clustering of spatial synergies, but the same procedure was followed also for clustering the temporal synergies. A schematic representation of this “functional similarity” clustering approach is given in Fig. 7. First, using the LDA approach we described above, for each spatial synergy of the *N* spatial synergies we computed the 72 × 72 confusion matrix showing how well it decodes the 72 performed movements. Then, as similar confusion matrices reveal similar coding of task parameters, we grouped synergies using as clustering measure the similarity of their confusion matrices. We reshaped each confusion matrix as a 5184-dimensional vector and computed the correlation coefficient (*r*_*i*,*j*_) between all such pairs of vectors (corresponding to pairs of temporal synergies) across all pairs of participants. This procedure yielded an *N* × *N* lower triangular matrix, each entry of which represents the functional similarity of each pair of synergies. Then, using *r*_*i*,*j*_ as distance measure, we input all synergies from all participants to an agglomerative hierarchical clustering algorithm^108^. The algorithm created a hierarchical cluster tree from all synergy pairs (Matlab function “linkage” with the “complete” distance method, i.e., using as distance between two clusters the furthest distance between all pairs of objects across the two clusters^109^). The hierarchical trees obtained for the temporal and spatial synergies are shown in Figure 4-top. To form clusters, we used a ‘cutoff’ value of 0.6 (0.5 for the temporal synergies). Visual inspection confirmed that these cutoffs split the respective trees into well-separated tree ‘branches’.

We represented each resulting cluster by the average of all synergies belonging to it. Subsequently, we examined whether each cluster contained synergies that had a distinct functional role. To do this, we computed the average confusion matrix of all synergies belonging to each cluster for decoding all task parameters. By means of the average confusion matrices, we determined the task parameters that are reliably decoded as well as the ones that are typically confused by each cluster.

### Data availability

All data are available upon reasonable request.
